# Influence of radio-grain priming on growth, antioxidant capacity, and yield of barley plants

**DOI:** 10.1016/j.btre.2022.e00724

**Published:** 2022-03-24

**Authors:** Hebat-Allah A. Hussein

**Affiliations:** a Botany and Microbiology Department, Faculty of Science (Girls Branch), Al Azhar University, Cairo, 11751, Egypt; b Biology Department, University College of Nairiyah, University of Hafr Al Batin (UHB), Nairiyah, 31991, Saudi Arabia

**Keywords:** Hordeum vulgar L., Gamma radiation, Lipid peroxidation, Antioxidant enzymes, Protein profile

## Abstract

The study aimed to examine the influence of different doses (0, 5, 10, and 20 Gray) of gamma radiation on growth, yield traits, and biochemical constituents of barley plants. The low doses (5 and 10 Gy) significantly improved the growth and yield of barley crop. Surprisingly, a higher dose (20 Gy) increased shoot growth and tillers number. Photosynthetic pigments were increased markedly at low doses (5 Gy) whereas decreased at high one. Gamma radiation enhanced total phenols, total flavonoids, total amino acids, antioxidant enzymes and H_2_O_2_. In addition, the protein profile showed varies in response depending on the applied dose. Conversely, gamma rays resulted in lower total sugars and proline than the corresponding control values. Ultimately, the modified antioxidant potential, protein pattern, and metabolic changes of barley exhibited the effectiveness of low doses of gamma irradiation in improving growth, and yield of barley plants.

## Highlights

• Ionizing radiation is a promising technology to improve crop productivity.
• Low doses of gamma radiation improved the growth and yield of barley plants.
• Proline in barley plants is susceptible to gamma rays.
• Gamma rays induced cross-take between free radicals and antioxidant systems.

## 1 Introduction

Barley (Hordeum vulgar L.) is considered one of the strategic cereal crops in the world involved Egypt. It occupies the fourth position in total cereal production after wheat, rice and maize [19]. It is utilized for many purposes like animal feeding, bread making, malting and mixing with wheat flour in some places [18]. In Egypt, the barley crop represents the main crops cultivated in coastal regions and the newly reclaimed lands under different irrigation systems [37]. Unfortunately, barley production decreased from 3.03 tons/ha in the 2000/2001 growing season to 1.55 tons/ha in 2015/2016 [19]. It is necessary to find potential techniques to improve plant growth and yield under sandy soil condition and climate changes.

Gamma irradiation represents a recent technology to improve the qualitative and quantitative attributes of many economical plant species [3, 16]. It solves many agricultural crop problems such as contamination, destruction of insect pests and microbial plant diseases resulted in postharvest losses [14, 23]. This technology induces differences in biological levels in many crops [52]. Moreover, it is a successful method in plant breeding programs. The magnitude effect of gamma (γ) rays depending on its dose and intensity [27].

All previous studies recommended further expanded research with other specified doses of *γ*-ray treatment of grains to improving barley productivity. Considering the role of ionizing radiation in solving many problems of different crops in Egypt, the current study aimed to find the optimum doses producing stimulation the growth and yield of barley plants grown under sandy soil conditions. It is done through observation the growth parameters, enzymes activity and protein profile.

## 2 Materials and methods

### 2.1 Experimental design

A greenhouse experiment was carried out during the winter season of 2017/2018 at the faculty of science (Girl Branch), Al-Azhar University, Nasr City, Cairo, Egypt. The study aimed to the effect of pre-treatment with low doses of γ- radiation on growth, biochemical constituents, and yield characters of barley plants (*Hordeum Vulgare* L.). Barley grains (Cultivar; Giza 125) were obtained from the Agriculture Research Center (ARC), Giza, Cairo, Egypt. The seeds were irradiated by gamma ^60^Co at different doses like 0 (non-irradiated), 5, 10, and 20 Gray (Gy) at the Egyptian Atomic Energy Authority (EAEA), Cairo, Egypt. Barley grains were sown on November 21th in earthenware pots No. 50 filled by sandy soil with six replicates for each treatment. The soil texture was sandy, field capacity 11.5%, pH 8.7, EC 0.35 dSm^−1^, Cl^−^ 1.7, HCO_3_^−^ 1.10, Na^+^ 1.2, *K* ^+^ 0.25, Ca^++^ 1.27%, and Mg^++^0.58 meq *L*^−1^. Phosphorus and potassium were applied before sowing at a rate of 6.0 and 3.0 g per pot of calcium superphosphate (15.5% P_2_O_5_) and potassium sulfate (48–50% K_2_O), respectively. Nitrogen was applied at the rate of 0.60 g per pot in the form of ammonium nitrate (33.5% N) after 30 and 60 days of sowing.

At 65 days after sowing, representative samples were taken from each treatment for determining the tested growth parameters; shoot length (cm), root length (cm), leaves No./ plant, flag leaf area as well as fresh and dry weights of shoot and root per plant. Tillers No./ plant, fertile spikes No./plant, spikes yield/ plant, grain yield/ plant, the 1000-grain weight (g), straw yield/plant (g) were determined at harvest time.

### 2.2 Determination of chemical constituents

Chemical constituents were estimated in the flag leaf at the vegetative stage.

#### 2.2.1 Photosynthetic pigments

Photosynthetic pigments; Chlorophyll a (Chl. a), Chlorophyll *b* (Chl. b) and total carotenoids were extracted from fresh leaves using 85% acetone and measured as detailed next. A 0.1 g fresh weight (FW) of barley flag leaves was homogenized with 85% acetone. The homogenized samples were centrifuged at 3000 rpm and the supernatant was made up to 10 ml with acetone (85%). The extract was measured at 663, 644 and 452 nm by spectrophotometer (VEB Carl Zeiss) using acetone as a blank. Pigment contents represented as mgg^−1^ FW using the following equations:

**Chlorophyll *a*** = 10.3 Ab_663_–0.918 Ab_644_ = µg/ml

**Chlorophyll *b*** = 19.7 Ab_644_–3.870 Ab_663_ = µg/ml

**Carotenoids** = 4.2 Ab_452_-(0.0264 chlorophyll *a* + 0.426 chlorophyll *b*) = µg/ml

#### 2.2.2 Total soluble sugars

Total soluble sugars (TSS) were determined in ethanol extract of dry flag leaves for barley plant by anthrone technique according to Cerning [9]. TSS were analyzed by reacting 0.1 ml of ethanol extract with 3.0 ml freshly prepared anthrone (150 mg anthrone + 100 ml 72% H_2_SO_4_) in a boiling water bath for 10 min and reading the cooled samples at 625 nm using spectrophotometer (VEB Carl Zeiss).

#### 2.2.3 Proline

To determine proline content in the tested plant samples, 0.5 g of fresh leaves was homogenized in 10 ml of aqueous sulfosalicylic acid (3%). Two ml of the filtrate was mixed with 2 ml of acid ninhydrin reagent and 2 ml of glacial acetic acid and remained for 1 h at 100 °C. After cooling, 4 ml of toluene was added to reaction mixture to extract the proline content and the absorbance was recorded by spectrophotometer (VEB Carl Zeiss) at 520 nm using toluene as a blank.

#### 2.2.4 Determination of total free amino acids

Total free amino acids in dry leaves of pre-irradiated barley plant were determined by ninhydrin method according to Rosen, [44] with some modifications. The ethanolic extract was prepared using 95% ethanol. After centrifugation, 1 ml of ethanolic extract was added to 0.5 ml buffer (20 ml distilled water, 5 ml glacial acetic acid to 27 g sodium acetate, 1.5 ml sodium cyanide (490 mg/L) and completed to 75 ml with distilled water (pH = 5.4). After that, 0.5 ml of ninhydrin solution (10 mg cadmium acetate in 0.2 ml glacial acetic acid, 0.8 ml distilled water, 200 mg ninhydrin, and the solution made up to 10 ml by 50% acetone) was added. Then, the mixture kept in a boiling water bath for 15 min. After cooling; 5 ml of 50% isopropanol was added. The purple color was measured against a reagent blank at 570 nm using a spectrophotometer (VEB Carl Zeiss). Free amino acids were calculated as mg/g dry weight using standard curve of l-glutamic acid.

#### 2.2.5 Total phenolics and flavonoids content

Total phenolics content in dry leaves was estimated by the method described by Savitree et al., [45] and Pourmorad et al., [43]. One ml of ethanolic extract was mixed with 10 drops of concentrated hydrochloric acid, heated rapidly in a boiling water bath for 10 min, cooled and 1 ml of Folin-Ciocalteau reagent and 1.5 ml of 14% sodium carbonate were added. The mixture was made up to 5 ml by distilled water, shaken well and then kept in a boiling water bath for 5 min. The absorbance at 650 nm was noted and the values represented as μg gallic acid equivalent (GAE) *g*^−1^ FW. However, total flavonoids content in dry leaves was determined according to method described by Adom and Liu, [2]. Appropriate dilutions of ethanol extract (2 ml) were mixed with 0.2 ml of 5% sodium nitrite, followed by 5 min, and then allow reacting with 0.2 ml of 10% aluminum chloride to form a flavonoid–aluminum complex. The absorbance was measured at 510 nm using the catechin as a standard.

#### 2.2.6 Assay of enzymes activity

##### 2.2.6.1 Enzymes extraction

The crud enzyme was extracted according to assay of different enzyme activities. A fresh leaf sample (2 g) was frozen in liquid nitrogen and finely ground by pestle in a chilled mortar, 10 ml of 100 mM phosphate buffer, pH 6.8 was added and incubated at 4   °C for overnight. After centrifugation at 5000 xg for 10 min, the supernatant was collected to assay the different enzymes activity [13].

#### 2.2.7 Peroxidase (POD, EC 1.11.1.7)

POD activity was assayed according to Darwesh et al., [15] with few modifications. A 0.2 ml of enzyme extract was added to buffer solution containing 5.8 ml of 50 mM phosphate buffer (pH 7.0), 2.0 ml of 20 mM pyrogallol and 2.0 ml of 20 mM H_2_O_2_. The increase in absorbance was determined within 60 s against a reagent without enzyme at 470 nm using spectrophotometer. One unit of enzyme activity is the amount of enzyme that catalyzed the conversion of one micromole of H_2_O_2_ per min at 25 °C [32].'

#### 2.2.8 Catalase (CAT, EC 1.11.1.6)

CAT activity was assayed according to the method of Chen et al., [10]. The reaction mixture with the final volume of 10 ml containing 40 µl of enzyme extract and 9.96 ml phosphate buffer (pH 7.0) containing H_2_O_2_ (0.16 ml of 30% H_2_O_2_ in 100 ml of 50 mM phosphate buffer) was prepared. CAT activity was determined by measuring the rate of H_2_O_2_ absorbance changing in minute against buffer blank at 250 nm using spectrophotometer. One unit of enzyme activity is defined as the amount of enzyme that reduced 50% of the H_2_O_2_ in 60 s at 25 °C.

#### 2.2.9 Hydrogen peroxide (H_2_O_2_)

H_2_O_2_ content determined using the method of Hussein et al., [25], in which fresh samples of leaf tissue (100 mg) was extracted with 5 ml of 0.1% trichloroacetic acid (TAC) and centrifuged at 12,000 g for 15 min. Then 0.5 ml of supernatant was mixed with 0.5 ml of 10 mM phosphate buffer (pH = 7) and 1 ml of 1 M potassium iodide. The absorbance was determined at 390 nm. The amount of H_2_O_2_, read using the extinction coefficient 0.28 µm^−1^ cm^−1^ and expressed as nmol *g*^−1^ FW.

### 2.3 Protein profile

The plant tissue (0.2 g) was rapid freezed with liquid nitrogen to make the plant more fragile and dried by mortar. The dried samples were mixed with 1 ml of water-soluble protein extraction buffer in eppendorf tube [12] and left in refrigerator overnight, and then vortexed for 15 s and centrifuged at 5000 xg at 4 °C for 15 min. The supernatants containing water-soluble proteins were transferred to new eppendorf tubes and kept at deep-freeze until use. To determine the relative molecular weight of extracted proteins, sodium dodecyl sulfate polyacrylamide gel electrophoresis (SDS-PAGE) was performed on a stacking and separating gel according to the method of Laemmli [33] using Mini-gel electrophoresis (BioRad, USA). The molecular weight of the isolated proteins was estimated in comparison to standard molecular weight markers (standard protein markers, 11–245 kDa; Sigma, USA). The protein bands were visualized by staining with Coomassie Brilliant Blue G-250 (Sigma, USA) after documentation [11].

### 2.4 Statistical analysis

Data of thirty measurements were analyzed through ± SD values by using SPSS statistics data document. The results were statistically analyzed according to [47]. The least significant differences (LSD) at 5% level of probability were calculated to compare the means of different treatments.

## 3 Results

### 3.1 Growth parameters

Data presented in Table 1 showed that the effect of γ-radiation low doses on barley growth parameters including; shoot length, No. of leaves, flag leaf area, fresh and dry weights of shoot per plant compared with the corresponding control values. Shoot length of irradiated plants (5 Gy) showed a significant increment reached 10% compared to control plants. However, the same growth parameter was decreased significantly (*P* < 0.05) in 10 or 20 Gy irradiated plant compared with control sample. The high dose (20 Gy) had the most depressing effect on shoot length of barley plants reached to 16.39%. Additionally, the leaves number was affected positively, especially at high doses of gamma irradiated plants at vegetative stage. The increment percent of leaves number reached 25.64% with 10 and 20 Gy. Gamma rays have variant effects on flag leaf area, the results showed that flag leaf area increased significantly with 10 Gy irradiated plants reached to 29.38%. However, high-applied dose (20 Gy) showed a depressing effect reached to 10% on flag leaf area compared to non-radiated plants. Furthermore, the results showed significant increases in fresh and dry weights of shoots for all *γ*-irradiated plants. Concerning the root parameters of barley plants showed varied response follow gamma radiation doses (Table 1**)**. The root length exhibited significant increases in 5 Gy or 10 Gy irradiated plants. The maximum increase (23.75%) was recorded in 10 Gy pretreated plants compared to control plants. Meanwhile, the reverse result was observed with 20 Gy gamma dose. However, irradiated plants showed non-significant response (P ˃ 0.05) in fresh and dry weights of the roots than those of control ones.

**Table 1:** Changes in growth parameters at vegetative stage of barley plants originated from gamma irradiated grains.

Gammaradiation	Shoot length (cm)	Root length(cm)	No. of leaves/ plant	Leaf area / plant(cm^2^)	Shoot fresh weight /plant(g)	Shoot dry weight / plant (g)	Root fresh weight / plant(g)	Root dry weight /plant (g)
Control	66.30 ± 1.5	16.0 ± 0.5	19.0 ± 1	24.0 ± 2.1	25.13 ± 1.1	2.70 ± 0.2	1.40 ± 0.2	0.31 ± 0.02
5 Gray	73.0 ± 2.0	19.0 ± 1.2	21.7 ± 2	31.1 ± 2.5	29.83 ± 2.5	3.22 ± 0.1	1.50 ± 0.4	0.39 ± 0.02
10 Gray	63.0 ± 2.1	19.8 ± 2.0	24.0 ± 2	34.93 ± 1.4	44.38 ± 1.1	4.50 ± 0.2	1.57 ± 0.3	0.40 ± 0.02
20 Gray	61.2 ± 1.2	14.5 ± 2.0	26.0 ± 2	27.0 ± 1.0	42.00 ± 1.5	4.2 ± 0.2	1.38 ± 0.1	0.34 ± 0.01
LSD% at 0.05	3.55	2.04	2.37	1.46	3.05	0.28	Ns	Ns

### 3.2 Yield components

Effects of γ-irradiation on yield components; tillers number, fertile spikes number, spikes yield/plant (g), grain yield/plant (g), 1000-grain weight (g), and straw yield/ plant (g) were studied in response to low doses of *γ*-radiation (Table 2). The tillers No., fertile spikes No. and weight of straw per plant showed significant and progressive increasing in *γ*-irradiated barley plants. The maximum increases reached 215.4, 60.0 and 192.1% for tillers number, fertile spikes number/plant, and weight of straw per plant, respectively of 20 Gy irradiated plants compared to the corresponding control values. Regarding spikes yield/plant, grain yield/plant and 1000-grain weight, the results showed significant variations (P < 0.05) depending on the applied dose. The maximum variations were observed with 10 Gy irradiated with maximum increases for the later mention parameters reached 43.82, 62.90 and 45.20%, respectively compared to the control ones.

**Table 2:** Changes in yield components of barley plants originated from gamma irradiated grains.

Treatments	Tillers no. /plant	Fertile spikes no. /plant	Spikes yield /plant(g)	Grain yield /plant(g)	1000-grain weight (g)	Straw yield/ plant(g)
Control	3.25 ± 0.5	2.00 ± 0.0	2.49 ± 0.97	2.21 ± 0.44	42.30 ± 3.40	4.18 ± 2.16
5 Gray	5.00 ± 0.8	4.00 ± 0.5	3.60 ± 0.23	3.16 ± 0.29	54.37 ± 3.45	9.27 ± 1.36
10 Gray	6.00 ± 2.1	4.00 ± 0.0	3.84 ± 0.59	3.60 ± 0.60	61.42 ± 2.67	11.00 ± 2.55
20 Gray	10.25 ± 1.8	3.00 ± 0.5	2.22 ± 0.26	2.11 ± 0.35	36.32 ± 2.51	19.49 ± 2.15
LSD% at 0.05	1.10	0.60	0.32	0.14	2.23	4.12

### 3.3 Photosynthetic pigments

**Fig. 1:**
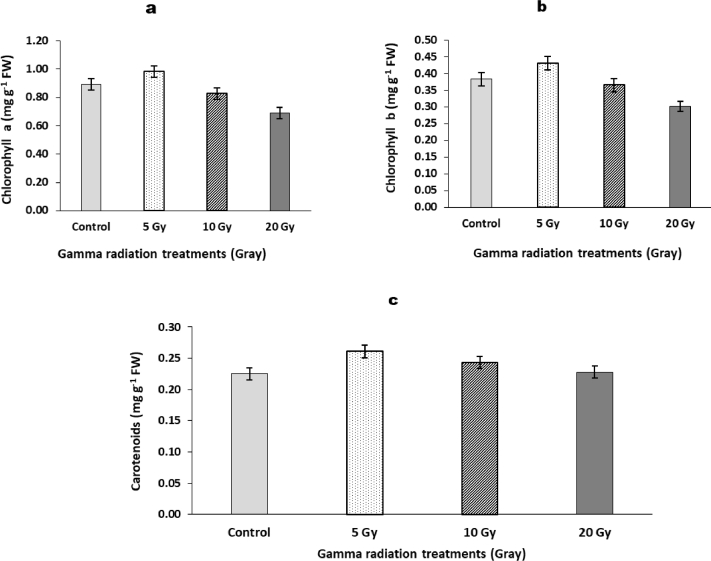
Changes in the photosynthetic pigments content in flag leaf of barley plants originated from gamma irradiated grains. LSD at *P* < 0.05 for chlorophyll *a* = 0.05 (a), chlorophyll *b* = 0.04 (b), Carotenoids = 0.02 (c), Vertical bars indicate ± SD.

Data represented in Fig. (1) showed the effects of γ-radiation pretreatments on photosynthetic pigments of barley plants. Only 5 Gy irradiated plants achieved increasing in chlorophyll "a" and "b" and carotenoids which reached 9.73, 12.27, and 16%, respectively, compared to those of non-radiated plants. Regarding to irradiated plants by 10 Gy, the results showed no significant change in photosynthetic pigments compared with control values. While, 20 Gy showed a marked decrease in the chlorophylls "a" and "b" but a non-significant change in carotenoids content compared to control values.

### 3.4 Total soluble sugars

**Fig. 2:**
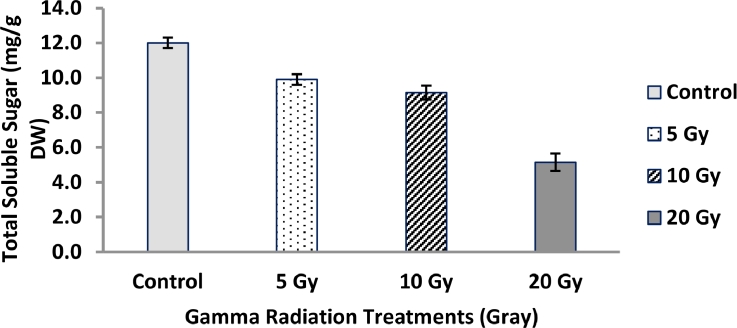
Changes in total soluble sugars content in flag leaf of barley plants originated from gamma irradiated grains. LSD at *P* < 0.05 = 0.64, Vertical bars indicate ± SD.

The results presented in Fig. (2) showed that pretreatments with gamma radiation resulted significant decrements (P ˂ 0.05) in total soluble sugar (TSS) in flag leaf of barley plants compared to the non-irradiated ones. The maximum decrement percentage reached 57.08% in leaves of barley plants raised form grains exposed to 20 Gy.

### 3.5 Total free amino acids and proline content

**Fig. 3:**
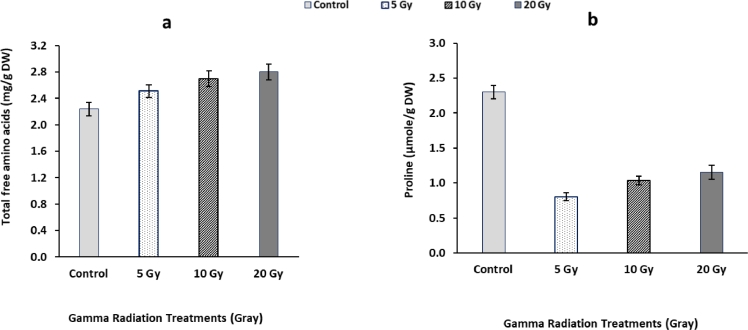
Changes in total free amino acids (a) and proline (b) contents in flag leaf of barley plants originated from gamma irradiated grains. LSD at *P* < 0.05 for total free amino acids = 0.05 (a), for proline = 0.17 (b), Vertical bars indicate ± SD.

Data represented in Fig. (3a) indicated that the pretreatment with low doses of gamma radiation caused progressive increment in total free amino acids content compared with that of the corresponding non-irradiated control plants. The maximum value of total free amino acids content was observed in 20 Gy irradiated plants and reached about 14.75% compared with control treatment. In case of proline contents, data illustrated in Fig. (3b) showed that the plants pretreated by low doses of γ-radiation caused significant decrement in proline contents. The maximum decrement was more obvious at the lowest applied dose (5 Gy) represented by 65% compared with control treatment.

**Fig. 4:**
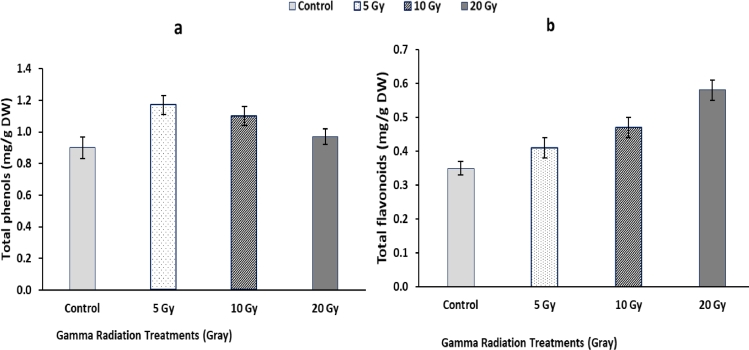
Changes in total phenols (a) and total flavonoids (b) contents in flag leaf of barley plants originated from gamma irradiated grains. LSD at *P* < 0.05 for total phenols 0.07, for total flavonoids 0.05, Vertical bars indicate ± SD.

### 3.6 Total phenols and flavonoids

The results in Fig. (4a) indicated that total phenols contents were increased significantly with low doses of gamma radiation pretreatments, especially at lowest dose (5 Gy) compared to that of the control value. The highest increment of phenolic content reached 30 and 22% by 5 and 10 Gy irradiated plants compared to the control value. Plants irradiated with (20 Gy) showed non-significant change in phenolic content compared to control value. For flavonoids content, data in Fig. (4b) indicated that total flavonoids content was increased gradually in flag leaf of barley plants with increasing gamma radiation dose. The maximum values were observed at 20 Gy represented by 65.71% compared to the control value.

### 3.7 Antioxidant enzymes

**Fig. 5:**
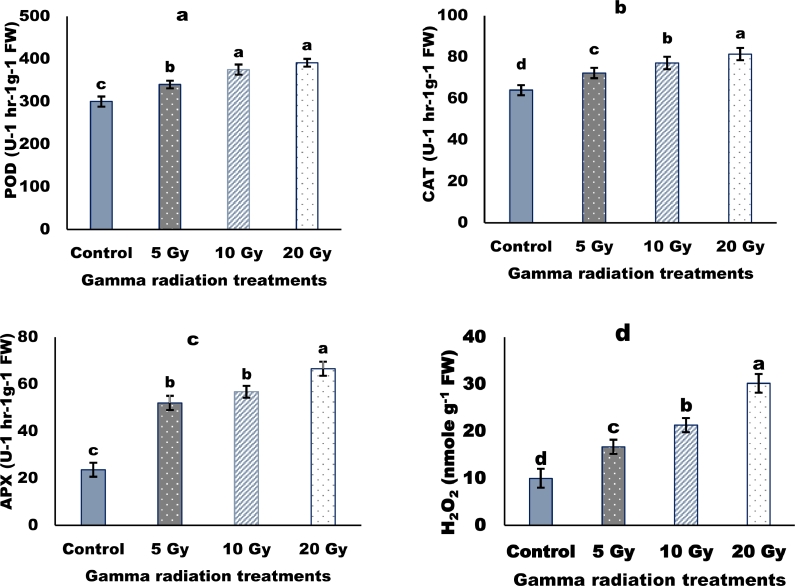
Changes in peroxidase activity (a), catalase activity (b), ascorbate peroxidase activity (c), and hydrogen peroxide content (d) in the flag leaf of *γ*-irradiated barley plants. *P* < 0.05, Vertical bars indicate ± SD.

The γ-irradiated barley plants showed a significant effect on peroxidase (POD), ascorbate peroxidase (APX) and catalase (CAT) activities (Fig. 5). Radiation treatments increased the activities of POD APX, and CAT enzymes. The highest increase in the 20 Gy-irradiated plants compared to the non-radiated plants. Also, gamma radiation pretreatments caused a progressive increase in hydrogen peroxide with increasing the radiation dose (Fig. 5d). The lowest value was in the control plant compared to other treatments.

### 3.8 Protein profile

**Fig. 6:**
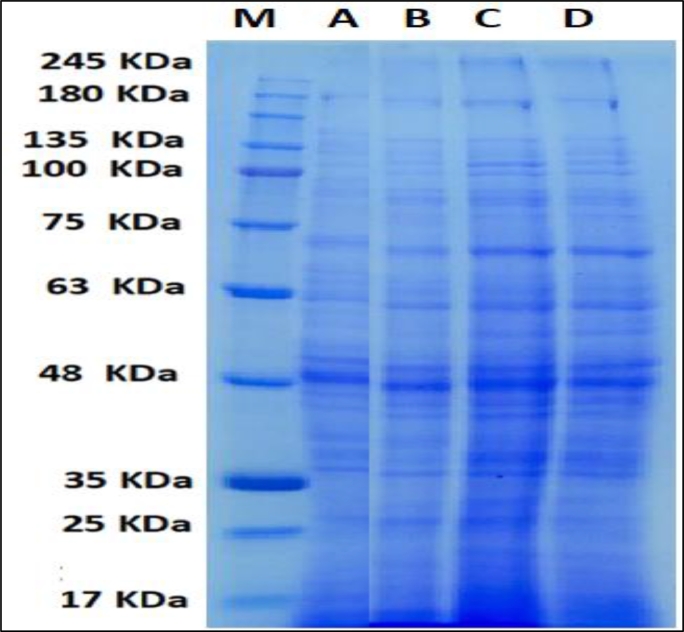
Changes in protein profile in barley plants originated from gamma irradiated grains. Where A, control; B, 5 Gray; C, 10 Gray; D, 20 Gray.

Comparison of the protein profile of irradiated barley plants revealed some differences in protein bands (Table 3 and Fig. 6). Gamma rays caused changes in protein patterns by inducing appearance and/or disappearance of some protein bands. Only at the low dose (5 Gy), the polypeptide with 65 kDa was induced. The irradiated plants with 5 Gy or 10 Gy induced formation of the polypeptide with 155 kDa. All tested doses of the radiation induced polypeptide with 19 kDa. On the other hand, only at the high dose (20 Gy), the polypeptide with 39 kDa was missed.

**Table 3:** Changes in protein profile in leaves of barley plants originated from irradiated grains.

No.	MW	Control	5 Gy	10 GY	20 Gy
1	170	+	+	+	+
2	155	–	+	+	–
3	110	+	+	+	+
4	100	+	+	+	+
5	89	+	+	+	+
6	78	+	+	+	+
7	72	+	+	+	+
8	70	+	+	+	+
9	68	+	+	+	+
10	67	+	+	+	+
11	65	–	+	–	–
12	59	+	+	+	+
13	53	+	+	+	+
14	50	+	+	+	+
15	47	+	+	+	+
16	44	+	+	+	+
17	42	+	+	+	+
18	39	+	+	+	–
19	37	+	+	+	+
20	35	+	+	+	+
21	34	+	+	+	+
22	33	+	+	+	+
23	30	+	+	+	+
24	28	+	+	+	+
25	27	+	+	+	+
26	23	+	+	+	+
27	21	+	+	+	+
28	20	+	+	+	+
29	19	–	+	+	+
30	18	+	+	+	+
31	17	+	+	+	+
32	15	+	+	+	+

## 4 Discussion

Barley (Hordeum vulgare L.) as a cereal plant and edible grain grow in a variety of environments. Incidentally with widely benefits, its productivity is decreased yearly. For that, it is crucial to apply new technologies for addressing planting problems and increasing the productivity [53]. The results declared that the potential stimulatory effects of irradiation at low doses on improving growth and yield of barley plants was investigated. Such stimulatory effects of gamma radiation on plant growth might be due to changing hormonal signaling network in plant cells [31, 40]. Additionally, low doses of γ-radiation could stimulate plant growth by altering leaf gas exchange, evaporation, enzymatic activities and improving crop yields [49]. Moreover, the growth stimulation of radiated barley plant might be attributed to activation of RNA and protein synthesis during the germination stage [23]. Furthermore, the biological effects of gamma radiation is mainly due to the formation of free radicals through the hydrolysis of water, which may result in the dilation of thylakoid membranes, alteration in photosynthesis, the modulation of an antioxidative system, accumulation of phenolic compounds and chlorophyll pigments [20]. However, the lower values of growth parameters in high dose irradiated barley plants might be due to the hormonal balance of endogenous ethylene and auxin activity directly in γ-radiated plants [35]. In this respect, Volkova et al., [51] reported that the pretreatment of barley grains with high gamma irradiation promoted expression changes in transcripts, DNA damage repair and antioxidant system.

Chlorophyll and carotenoids represent the main part of the green plants energy, therefore, any alteration in their contents causes parallel effects on the plant metabolism [26]. Data in the present study indicated that photosynthetic pigments increased only in barley plants irradiated with the lowest dose (5 Gy). These results agree with the obtained results by Kim et al., [30] who found that gamma radiation at 16 Gy increased photosynthetic pigments of red pepper plants. An increase in chlorophyll pigments may be attributed to radiation effect on activation of certain chloroplast enzyme systems involved in the synthesis of photosynthetic pigments [28]. However, the maintenance of the photosynthetic pigments after irradiation at 10 Gy might be due to adjusting the redox metabolism that represented a regulatory process at the growth stage [42, 51]. On the other side, gamma radiation at 20 Gy resulted in negative effects on photosynthetic pigments in barley plant. These results could be explained by gamma rays induce certain oxidative stress which causing lipid peroxidation of chloroplast membrane leads to chlorophyll degradation. At the same time, high dose of radiation increases the activity of peroxidase enzyme leads to protect the chloroplast apparatus.

The reduction of total soluble sugars might be representing a regulating response in photosynthesis process and adjustment homeostasis in gamma irradiated plants [17]. These led us to conclude that gamma radiation influences sugar-starch inter-conversion that in turn to change total carbohydrates content and improve plant growth. The accumulation of total free amino acids in gamma irradiated plants might be due to maintain osmotic potential in the vacuoles and cytoplasm at an optimal level of cell metabolism, hence protected cell structures from free radicals. Additionally, the results in declared that there are a strong correlation between the low levels of total soluble sugars and high levels of total free amino acids. Such a correlation might be due to the conversation of glucose into aromatic amino acids through the shikimate pathway. These suggested that low dose of gamma radiation might act as an activator for amino acids biosynthesis which in turn to change protein content that have essential roles in plant growth [50]. On the contrary, gamma radiation leads to a marked decrease in proline content as compared to control value. These results are harmony with those of Auda & AL-Wandawi, [5] who worked on Iraqi dates (Phoenix dactylifera L.) exposed to gamma radiation treatments. The results proved that proline in barley plants is a sensitive amino acid to *γ*-radiation treatments.

Phenols are considered the cooperative network, using a chain of several redox reactions. Total phenols contents were increased with 5 and 10 Gy irradiation dose. The significant positive correlation between low dose of gamma radiation and total phenols contents can help to induce protective mechanisms against the membrane damages. These results are harmony with Adamo et al., [1] who suggested that *γ*-radiation resulted in oxidative stress leading to breaking the biochemical bonds of polyphenols, releasing low molecular weight soluble phenols. Additionally, our results suggested that the role of gamma radiation might be linked to activation of antioxidant enzymes, which control phenolic compounds level. Application of gamma radiation induced the accumulation of total flavonoids in barley plants. These results are similar to the study of Hanafy & Akladious, [23] on fenugreek plants, γ-radiation generates free radicals that could act as stressor signals promoting the synthesis of flavonoid compounds with high antioxidant properties [8]. Moreover, the biosynthesis of flavonoids in the plant might be due to their antioxidant and protective roles in plant growth and development.

POD and CAT are enzymes stimulate the diversion of H2O2 to water and O2 [21]. The balance between ROS generation and activities of antioxidant enzymes determine whether oxidative signaling and/or damage will occur [39]. Treatment with gamma radiation results increment of POD activity but decrement of CAT activity. In this concern, Jan et al.  [27] reported that gamma radiation increase antioxidant enzymes. Enzyme activation may be due to a modulatory effect of *γ*-radiation on enzyme structure [29]. The higher POD, APX and CAT enzymes activities represent a pointer to the H2O2 split. It suggests that the change in the activities of these antioxidant enzymes might be due to their modulated role in protective processes against the oxidative damage resulting from gamma radiation. In the current study, high irradiation dose resulted in a significant increase in H2O2 content compared to other treatments. The increment in H2O2 level depending on *γ*-radiation dose might be due to stimulating and accumulating the free radicals leading to oxidative toxicity [48].

Exposure of the seeds to low doses of gamma rays induced the synthesis of new proteins in barley plants. These results are similar to the study of Hameed et al., [22] on chickpea and fenugreek plants, respectively. The synthesis of new protein is one of the protective mechanisms of plant in response to gamma irradiation [4]. Moreover, the effect of *γ*-radiation proteins synthesis might be due to the formation of the disulfide bond between polypeptide chain leading to the aggregation and conformation of the low molecular weight protein [23]. On the other hand, the depletion of some proteins with high dose of gamma rays may be due to higher metabolic and hydrolyzing enzyme activities [36] and/or to disturbance of the protein synthesis leading to incorrect folding and damage protein structure [22].

## 5 Conclusion

Gamma radiation at low doses stimulates photosynthetic pigments, proteins, free amino acids, flavonoids, phenolic compounds, the cross-take between hydrogen peroxide and antioxidant enzymes; peroxidase, ascorbate peroxidase, and catalase. Hence, the biochemical changes participate in improving growth and increasing yield.

## Funding

This research did not receive any specific grant from funding agencies in the public, commercial, or not-for-profit sectors.

## Declaration of Competing Interest

The author declare no conflicts of interest.
